# First evaluation of the NHS Direct Online Clinical Enquiry Service: A Nurse-led Web Chat Triage Service for the Public

**DOI:** 10.2196/jmir.6.2.e17

**Published:** 2004-06-02

**Authors:** Nina Eminovic, Jeremy C Wyatt, Aideen M Tarpey, Gerard Murray, Grant J Ingrams

**Affiliations:** ^1^Academic Medical CentreDepartment of Medical InformaticsAmsterdamThe Netherlands; ^2^Research and Development ProgrammeNational Institute for Clinical ExcellenceLondonUK; ^3^NHS Direct OnlineSouthamptonUK; ^4^The Crossley PracticeCoventryUK

**Keywords:** E-health, telemedicine, Web chat, triage, NHS Direct Online

## Abstract

**Background:**

NHS Direct is a telephone triage service used by the UK public to contact a nurse for any kind of health problem. NHS Direct Online (NHSDO) extends NHS Direct, allowing the telephone to be replaced by the Internet, and introducing new opportunities for informing patients about their health. One NHSDO service under development is the Clinical Enquiry Service (CES), which uses Web chat as the communication medium.

**Objective:**

To identify the opportunities and possible risks of such a service by exploring its safety, feasibility, and patient perceptions about using Web chat to contact a nurse.

**Methods:**

During a six-day pilot performed in an inner-city general practice in Coventry, non-urgent patients attending their GP were asked to test the service. After filling out three Web forms, patients used a simple Web chat application to communicate with trained NHS Direct triage nurses, who responded with appropriate triage advice. All patients were seen by their GP immediately after using the Web chat service. Safety was explored by comparing the nurse triage end point with the GP's recommended end point. In order to check the feasibility of the service, we measured the duration of the chat session. Patient perceptions were measured before and after using the service through a modified Telemedicine Perception Questionnaire (TMPQ) instrument. All patients were observed by a researcher who captured any comments and, if necessary, to assisted with the process.

**Results:**

A total of 25 patients (mean age 48 years; 57% female) agreed to participate in the study. An exact match between the nurse and the GP end point was found in 45% (10/22) of cases. In two cases, the CES nurse proposed a less urgent end point than the GP. The median duration of Web chat sessions was 30 minutes, twice the median for NHS Direct telephone calls for 360 patients with similar presenting problems. There was a significant improvement in patients' perception of CES after using the service (mean pre-test TMPQ score 44/60, post-test 49/60; p=0.008 (2-tailed)). Patients volunteered several potential advantages of CES, such as the ability to re-read the answers from the nurse. Patients consider CES a useful addition to regular care, but not a replacement for it.

**Conclusions:**

Based on this pilot, we can conclude that CES was sufficiently safe to continue piloting, but in order to make further judgments about safety, more tests with urgent cases should be performed. The Web chat sessions as conducted were too long and therefore too expensive to be sustainable in the NHS. However, the positive reaction from patients and the potential of CES for specific patient groups (the deaf, shy, or socially isolated) encourage us to continue with piloting such innovative communication methods with the public.

## Introduction

NHS Direct is a national service founded in 1998 by the UK National Health Service (NHS) to provide a nurse-led 24-hour help and advice service over the telephone. NHS Direct is a free service (only the local telephone call costs) for the public [1] currently receiving approximately 7.5 million calls a year in England and Wales [2,3]. In 2000, the UK Government introduced a plan for future investment and reform within the NHS [4]. At the heart of these changes was the desire to provide a health service built around patients' needs, including the need for knowledge and information. Modern means of communication, such as the Internet, have introduced new ways for accessing health services and information. As in other, especially Western, countries the number of Internet users in UK is growing rapidly. NHS Direct decided to extend its services to the Internet, introducing NHS Direct Online in 1999 [5]. The Web site offers information about illness, keeping healthy, and how to access local health services. One of the functions of this Web site is an e-mail online enquiry service that offers more detailed information on health issues, but does not accept enquiries from patients about specific symptoms. Although this restriction is clearly stated on the Web site, nearly a quarter of all online enquiries are about symptoms [6]. This highlighted the need for a more personalized, clinical problem-based service.

With this in mind, NHS Direct Online developed a prototype Clinical Enquiry Service (CES) offering a secure, confidential one-to-one Web-based consultation with a nurse. It was thought that such a service might also provide access to health information and advice for clients whose needs are not currently met by the telephone NHS Direct service because of accessibility problems, e.g. the socially isolated or those with hearing or speech limitations.

There are various Web sites offering chat room services for specific patient groups, such as cancer patients [7] or schizophrenia patients [8]. A number of Web sites have scheduled chat sessions where patients can ask questions directly of a specialized physician in the field. However, there are no services offering a one-to-one chat service with a medical professional.The pilot study described here was set up to identify the challenges and implications of such a Web-based chat service. The aim of this pilot study was to explore for CES the three most important aspects of any eHealth service or application: its safety, its feasibility, and patient perceptions of it.

## Methods

### Procedure and Participants

The six-day pilot was performed in an inner-city general practice in Coventry (England), an industrial city. Non-urgent patients (ie, those telephoning the practice who did not request an appointment that day) were asked to participate in the study. There were no other inclusion criteria for the patients and their level of computer literacy was not a factor. When patients arrived in the waiting room, a researcher gave them a short explanation of the study and they were invited to participate and sign a consent form. The CES Web chat session took place soon after in an examination room. A researcher was present to assist the patient only if he or she needed assistance to proceed with the CES consultation, and to observe the patient's behavior and reactions. The patients could remain anonymous during the Web chat, but, for a more personal approach, provided a first name for the nurse to use. The first step in the chat was to exclude any urgent conditions by querying the patient about the presence of chest pain, shortness of breath, etc.

Five NHS Direct-trained nurses based in Southampton were further trained in the use of Web chat. These nurses used the same NHS Clinical Assessment System (NHS CAS), a triage decision support system generating questions and advice based on patient answers, used in the telephone NHS Direct service. The CES Web chat application could not be implemented on the same computer as CAS, so the nurses had to use two computers during each Web chat session.

The possible triage endpoints generated by CAS, which correspond to the advice that NHS Direct nurses give patients, were:

- call 999 for an urgent ambulance
- visit the accident and emergency department as soon as possible
- visit the accident and emergency department within 4 hours
- contact your GP within 4, 12, or 36 hours or within 2 weeks
- home care.

Immediately after participating in the CES Web chat session, all patients were seen by a GP at the same practice. The patients were instructed not to discuss the Web chat or the suggested end point with this doctor. A system manager was present at the practice to solve technical problems if they arose.

### The Web Chat Application

A Web chat application service was leased from Instant Service USA and was made accessible only to the nurses and patients participating in the pilot. The interface to the service was tailored to our needs. Prior to each Web chat session, patients filled out several online forms about their general health. The Web chat was between patient and nurse, and the discussion was not shared with anyone else. A log file of each session was stored on a secure server.

### Evaluation of Safety

One of the most important aspects of any innovation in health care is its safety. Prior to developing any telemedicine or eHealth service, it is important to decide exactly who the intended users are. It is equally important to decide who should not use the service (eg, very sick patients), and to test the service's ability to detect these patients to ensure its safe operation. In addition, we should check whether the service may harm eligible users included in the pilot study. In this pilot study, the safety of the Web chat was evaluated on two levels: the ability of the NHS CAS-assisted nurse to detect urgent cases, and agreement between the CES endpoint and the GP endpoint. After every consultation, and with the benefit of a full consultation, the GP recorded the advice he or she would have given to the patient if the patient had phoned the GP that day before attending, using the same list of possible endpoints as the Web chat nurse. For each patient, we compared the endpoint allocated by the GP with the endpoint allocated by the nurse at the end of the CES session. To validate the GP end point, the GPs also provided a list of interventions performed on all patients collected from the patient records, and patient outcomes after a 3-month follow-up.

### Evaluation of Feasibility

The evaluation of feasibility needs to include a check that that the resources required are likely to be available and that the technology has adequate coverage and some promise of cost-effectiveness. One of the common problems of telemedicine or eHealth applications is that they often involve not only expensive technology (video-conferencing tools, digital cameras, fast Internet connections), but also significant time, training, and changes in work practice for health-care professionals (8)]. We explored the general feasibility of CES by focusing on the range of potential CES users, the resources needed to run the service, and the quality of interpersonal communication. For all patients, age, gender, and self-reported computer literacy were recorded. In order to calculate the duration of the Web chat and its components, the intervals between specific defined events occurring during a CES session were logged into a file.

We compared the total duration of the Web chat session with the median duration of NHS Direct telephone consultations for patients calling with the same symptom. For each presenting symptom, the median call duration of a random sample of 30 cases with the same symptoms was calculated by NHS Direct.

Since communication using Web chat consists only of exchange of typed text, we explored whether this was enough to establish a sufficient level of rapport between the nurse and patient. Patients were interviewed about this, and the log files were used to retrieve any nurse questions for which the patient response indicated a need for clarification or rephrasing.

### Evaluation of Patient Perceptions

Patient perceptions about a variety of issues were checked using three instruments. The Telemedicine Perception Questionnaire (TMPQ) [9], a validated questionnaire designed by the University of Minnesota, was used to measure the change in patient perception of CES after using it. The questionnaire was adapted to our study with permission by slight rephrasing (changing "home telecare" into "CES" or "Web chat") or excluding some questions (eg, those about costs). The users scored each item twice: before and after using CES, with each score ranging from 1 (strongly disagree) to 5 (strongly agree). The highest possible score with our modified TMPQ was 60. Some items (*italic* in Table 3) were negatively worded, so scores for these items were transformed (eg, a score of 5 was transformed into 1) during the analysis, so that higher scores always represent positive patient attitudes toward CES. A paired sample t-test was used to check for differences between the pre- and post- test scores. We also used a second, researcher-administrated, instrument containing 23 open and closed questions to capture patient comments and experiences with CES. Finally, all patients were observed by a researcher to study their behaviour and log revealing comments spontaneously made by the patients during the Web chat.

Data analysis was performed using SPSS 10.1.

## Results

### Safety

The nurses were instructed to ask several questions in order to exclude any possibility of an urgent problem. However, the patient could have shown signs of urgency without the nurse responding to them. The log file analysis showed that the nurse responded to every patient comment or question. This was partially enforced by the Web chat application, as the nurse could see when the patient was typing, which prevented the nurse and patient typing at the same time.

The GP consultation resulted in 13 patients receiving advice only, 13 a prescription, three investigations, two referrals and one a medical certificate. Eight patients received multiple interventions. More than half (57%) the patients did not go back to their GP for the problem, and 30% (7/23) returned only once during the three-month follow-up period. Three patients (13%) returned more times (three, four, and eight times) to their GP for the following problems: urinary frequency, chest pain after taking medicine for acid indigestion, and carcinoma of the prostate.

**Table 1:** Agreement between CES and GP endpoints

GP endpoint					Total	Match
	Contact GP within 12 hours	Contact GP within 36 hours	Contact GP within 2 weeks	Self-care		
Contact GP within 12 hours	4	1	5	0	10	40%
Contact GP within 36 hours	1	1	3	1	6	17%
Contact GP within 2 weeks	0	1	4	0	5	80%
Self care	0	0	0	1	1	100%
Total	5	3	12	2	22*	46%

^*^ One patient did not finish the Web chat session due to lack of time.

We found an exact match between the CES endpoint and the endpoint defined by the GP in 45% (10/22) of patients (Kappa (Κ)=0.25; 95% confidence interval 0.37 to 0.45, which is low) [10]. Where there was a difference in endpoints, in most cases the CES nurse suggested a more urgent follow-up than the GP (45%; 10/22). With only two patients did the CES nurse propose a less urgent consultation (shown as bold in Table 1). These two patients attended their GP for acute lumbar back pain and tiredness.

### Feasibility

During the pilot, 25 patients agreed to participate. Two patients were excluded from further analysis as they were members of the practice staff. More than half (57%, 13/23) of the patients were female. Patients varied in age (range 19-80, mean 48 years), educational background, and occupation. Although 78% (18/23) of patients considered themselves computer literate, only 64% (14/22) reported good typing abilities. Not all patients were experienced PC users, but 87% (20/23) found it easy to describe their symptoms on the Web, and 96% (22/23) stated that CES offered sufficient rapport with the nurse. No patient asked for a clarification of any of the questions asked by the nurse.

The Web chat connection with the nurse was disconnected four times in 25 consultations, but each time was easily re-established.

The median duration of the Web chat sessions was 30 minutes [25th percentile 23, 75th percentile 36 minutes]. This was more than twice as long as for a similar group of patients using the telephone NHS Direct services (Table 2). There was a positive correlation between patient age and total duration of Web chat (*r_s_*=0.44, p=0.04 (2-tailed)).

Almost all patients (96%, 21/22) were happy about the time that the Web chat took to complete. One patient disconnected himself after 10 minutes as the Web chat took longer than he expected and a technical problem occurred.

**Table 2:** Comparison of session duration for CES and the telephone NHS Direct service

CES problem	CAS code	NHS Direct duration	CES duration	Difference*
Painful left elbow	arm injury	0:13:27	0:31:39	0:18:12
Acute lumbar back pain	back pain	0:12:09	0:22:51	0:10:42
Back pain			0:40:32	0:28:23
Acute lumbar back pain			0:28:56	0:16:47
Lumpy area in both breasts	breast lump	0:10:54	0:29:39	0:18:45
Chest pain after taking medicine for acid indigestion	chest pain	0:11:49	0:46:04	0:34:15
Prolonged cough	cough	0:15:22	0:49:04	0:33:42
Chronic cough			0:23:48	0:08:26
Cough and chest infection			0:29:01	0:13:39
Impacted ear wax	ear problems	0:12:27	0:32:49	0:20:22
Lethargy/tiredness	fatigue	0:13:30	0:17:33	0:04:03
Knee pain	knee pain/swelling	0:11:50	0:19:53	0:08:03
Recovery advice after knee cartilage operation			0:39:25	0:27:35
Whiplash after car accident	neck injury	0:12:03	0:35:26	0:23:23
Contact dermatitis on hands	rash	0:14:59	0:40:02	0:25:03
			0:34:53	0:19:54
Itchy face and neck				
			0:17:43	0:02:44
Dry and discolored nails				
			0:22:59	0:08:00
Intertriginous rash				
Tonsillitis	sore throat	0:14:56	0:31:39	0:16:43
Frequent urinating	urine frequency	0:14:50	0:25:24	0:10:34
Review of polymyalgia/frequent urinating			0:29:23	0:14:33
			0:33:08	0:18:18
Waterworks problems/review of post- operative ovarian cyst.				
**Overall (medians)**		**00:12:57**	**00:30:39**	**00:17:29**

^*^ Session duration is in minutes. Comparison is of cases with similar clinical problem (median of 30 randomly selected cases).

Turning to the components of the CES consultation, the first part of each session was exclusion of any urgent conditions and this took a median of 9 minutes [25th percentile 6, 75th percentile 11 minutes]. This process also included answering several questions on a Web form prior to the Web chat. The median duration of discussion about the patient's current problem was 18 minutes [25th percentile 9, 75th percentile 25 minutes].

### Patient Perceptions

The highest mean score (3.9) in the TMPQ pre-test was for patients' estimate of the ability of nurses to obtain a good understanding of their health problem over the Internet; the lowest was 3.2 for concern about the lack of face-to-face contact. The most positive opinion following experience with CES was for CES as an addition to regular care (4.3). A paired sample t-test was performed with 20 cases, as one pre-test answer and two post-test answers were missing. The analysis showed significantly higher mean post-test scores compared to pre-test scores (mean pre score 44/60, mean post 49/60, paired sample t-test: p=0.008 (2-tailed), score difference 5, (95%CI 7.4-1.3)). It is unlikely that the missing data would significantly alter the mean score for these items. Patient perceptions improved for all items after using the CES Web chat (Table 3), but this improvement was significant for only two items, about CES becoming a standard way of health assessment in the future and CES making it easier to contact NHS Direct.

**Table 3:** Adapted TMPQ with mean scores for pre- and post-test for 20 patients *

Question	Pre- mean	Post- mean	Mean difference	2-tailed p-value
A nurse can get a good understanding of my health problem over the Internet.	3.9	4	0.1	0.9
*I am concerned that the NHS Direct Online Clinical Enquiry Service (CES) is a threat to my privacy.*	3.7	4.1	0.4	0.16
*The use of a personal computer seems difficult or unreliable to me.*	3.8	4.2	0.4	0.23
I can be as satisfied talking to a nurse over the Internet as talking in person.	3.5	4	0.5	0.15
The NHS Direct Online CES can improve my understanding of my health.	3.8	4.2	0.4	0.09
*I am concerned that there is no face-to-face conversation during the use of the NHS Direct Online CES.*	3.2	3.7	0.5	0.14
The NHS Direct Online CES is a convenient form of health assessment for me.	3.8	4.2	0.4	0.11
The NHS Direct Online CES will save me time.	3.8	3.9	0.1	0.82
**The NHS Direct Online CES will be a standard way of health assessment in the future.**	**3.7**	**4.2**	**0.5**	**0.014**
The NHS Direct Online CES can be an addition to the regular care I receive.	4	4.3	0.3	0.08
*A nurse cannot assess me as well over the Internet as in person.*	3.3	3.7	0.4	0.2
**The NHS Direct Online CES makes it easier for me to contact NHS Direct.**	**3.7**	**4.2**	**0.5**	**0.004**
**Overall mean score**	**3.7**	**4**	**0.3**	**0.008**

^*^ Significant changes are in bold. Italicized items are negatively worded items for which the responses were re-coded.

The total TMPQ score per individual patient decreased after using CES for four patients (ranging from 7 to 1 points less than in pre-test, mean fall 3.8) and increased for 15 patients (ranging from 1 to 18 points more than in pre-test, mean rise 6.8). One patient did not change her opinion about CES after using it. No correlation was found between patient age or gender and perception about CES measured using TMPQ.

## Discussion

The results of this pilot suggest that the CES nurse-led Web chat service might be safe as a triage system for non-urgent patients. This safety aspect is supported by the fact that CES nurses suggested a more urgent follow-up than the GP for the same symptoms in almost half of the cases. However, patients who participated in this pilot study were not typical users of CES, as they had already made a decision to visit their GP. Although a general practice as study setting is a safe and practical environment for the first pilot study, further studies should be performed in a home or workplace setting where the service would actually be used by the public. CES was designed to for members of the public who are hard to reach by other means of communication. In this first pilot, we did not present the service to this group of users.

Exclusion of urgent patients is one of the important functionalities of CES. Because we did not include urgent patients in our study, we are not able to judge whether CES would be a safe and reliable service in a real life situation.

As all communication between the nurse and patient is based on typed text, signs of emotion and empathy are hard to communicate. Therefore, it is possible that some answers given by patients, and some questions asked by nurses, might be interpreted differently than expected. Another threat of such indirect communication is the possibility that patients might give untrue information (eg, incorrect details of their age, sex, severity of symptoms, or geographical location) that cannot be checked by the nurse.

Patients with a wide range of age, gender, and computer experience were able to explain their symptoms using Web chat and to establish an adequate level of communication with the CES nurse. The Web chat of older patients lasted longer than that of younger patients. It is difficult to determine for which age this difference is significant as we had small samples from each age group. This difference might be due to the patients' speed of typing. Since we did not succeed in finding a similar study exploring one-to-one Web chat between a patient and health professional, we compared the duration of CES triage with the duration of telephone triage. In general, CES Web chat took twice as much of the nurse's time as NHS Direct telephone triage and this was considered unacceptable from the financial point of view. However, we believe that this problem is partly due to an inefficient information management system (eg, CAS and CES were running on two different computers) and time-consuming procedures, such as exclusion of emergency conditions by the nurse. Most patients did not have problems with the duration of a CES session.

Although CES would be too expensive as a service open to any UK patient in the same way as NHS Direct, it might be helpful for certain groups of patients, such as those who wish to discuss private problems (eg, sexually transmitted diseases, HIV, psychological problems, addiction) [11- 13], especially where they can be overheard, such as at work or at home, and for people with speech problems (eg, those who are deaf, shy, have dysphasia or other types of speech difficulties).

The TMPQ is a validated instrument, but it underwent some adjustments before using in the pilot and was not re-validated. In further studies, this adapted questionnaire should be re-validated. Nevertheless, patients were positive about the potential of a Web chat version of NHS Direct and became even more positive after trying out the service, independent of their age and gender. After using CES, some patients stated that this service will become a standard way of health assessment in the future. However, as NHS Direct Online intended, most patients considered CES an addition to regular care, not a replacement for it. Privacy issues did not seem to be a problem for the patients included in our study. A significant improvement in patient perception was found about the ease of using CES to contact NHS Direct. The patients were more positive about the service after having tried it out. The technology became less "scary" after using the service, and patients started to recognize the advantage of CES over the telephone service. Several patients volunteered novel benefits of the Web chat medium over the telephone NHS Direct service, such as the ability to re-read the nurse's answers and having more time to think about her questions before responding. An 80-year old patient described these advantages as follows:

...When you go to a doctor, you forget things because you're a little bit nervous. You forget what you've said, to say what you wanted to say and what has been said by the doctor. Using this service you have more time to think and ask anything you want, you can see what you and the nurse said, that is much better...

## Conclusions

This study was performed to explore whether the pilot CES service was sufficiently safe, feasible, and acceptable to patients to justify a larger study. We can conclude that CES was sufficiently safe in the pilot phase, but in order to make further judgments about safety, more testing with urgent cases should be performed. At this stage, further development on the CES service has been postponed because of the long duration of conversation between the patient and nurse, and other NHS Direct Online priorities. The pilot resulted in several recommendations about how to improve the software and decrease the communication time. Communicating with patients through the Internet should first be further explored in more closed, controlled settings, such as GP practices or health centres, before this service is offered to the public at large. However, it is likely that commercial companies will develop and offer such services before scientific studies are performed. Therefore, we believe that early pilots such as ours, exploring safety, feasibility, and acceptability, are important to predict the risks and benefits of eHealth applications such as CES.

## Abbreviations

NHS: National Health Service
NHSDO: National Health Service Direct Online
CAS: Clinical Assessment System
CES: Clinical Enquiry Service
NHSCAS: National Health Service Clinical Assessment System
SPSS: Statistical Package for the Social Sciences
TMPQ: Telemedicine Perception Questionnaire
